# Simultaneous Production of Biosurfactants and Bacteriocins by Probiotic *Lactobacillus casei* MRTL3

**DOI:** 10.1155/2014/698713

**Published:** 2014-01-29

**Authors:** Deepansh Sharma, Baljeet Singh Saharan

**Affiliations:** Microbial Resource Technology Laboratory, Department of Microbiology, Kurukshetra University, Kurukshetra, Haryana 136 119, India

## Abstract

Lactic acid bacteria (LAB) are ubiquitous and well-known commensal bacteria in the human and animal microflora. LAB are extensively studied and used in a variety of industrial and food fermentations. They are widely used for humans and animals as adjuvants, probiotic formulation, and dietary supplements and in other food fermentation applications. In the present investigation, LAB were isolated from raw milk samples collected from local dairy farms of Haryana, India. Further, the isolates were screened for simultaneous production of biosurfactants and bacteriocins. Biosurfactant produced was found to be a mixture of lipid and sugar similar to glycolipids. The bacteriocin obtained was found to be heat stable (5 min at 100°C). Further, DNA of the strain was extracted and amplified by the 16S rRNA sequencing using universal primers. The isolate *Lactobacillus casei* MRTL3 was found to be a potent biosurfactant and bacteriocin producer. It seems to have huge potential for food industry as a biopreservative and/or food ingredient.

## 1. Introduction

Probiotics are living microbial preparations that have beneficial effects on the well-being of the host when administered in adequate amount [1]. A range of beneficial effects have been reported for probiotics, including improvement in digestion [2], antidiarrheal property [3], and prevention of food-borne pathogens [4]. Food-borne pathogens such as *Listeria monocytogenes*, *L. innocua*, *Pseudomonas aeruginosa*, *Salmonella typhi*, *Staphylococcus aureus*,* S. epidermidis*, and *Bacillus cereus* not only are harmful to human health, but also lead to spoilage of foodstuff. Although chemical preservatives efficiently inhibit the growth of food-borne pathogens, their use in food industry is still under scrutiny, as some of them have been reported to be unsafe to human health and for the environment. LAB produce a large number of antimicrobial compounds such as organic acids, H_2_O_2_, diacetyl, enzymes, bacteriocins, and biosurfactants which are effective against food spoilage and pathogenic bacteria. Amongst these antimicrobial metabolites, bacteriocins have been found as a potentially safe class of biopreservatives. For example, nisin, a bacteriocin produced by *Lactococcus lactis*, has been used since last decade to extend the shelf  life of preserved food [5, 6]. The bacteriocins are latent biopreservatives, particularly in meat, packaged food, and dairy products [7]. A large number of reports indicate the potential use of bacteriocins in the control of important gastric pathogens specially *Salmonella* sp. [8], *Campylobacter jejuni*, *L. monocytogenes* [9], and *E. coli* O157:H7 [10].

Microbial biosurfactants are amphiphilic metabolites with a pronounced surface activity with a broad range of chemical structures (such as glycolipids, lipopeptides, polysaccharide-protein complexes, phospholipids, fatty acids, and neutral lipids) with several advantages over chemical surfactants, that is, low toxicity, biodegradable, and effective at different ranges of temperature and pH [11, 12]. They are being used for industrial applications in the pharmaceuticals, biomedical, and food processing industries [11, 13, 14]. This paper deals with the study of the biotechnological potential of *strain L. casei *MRTL3. The selective potential of the strain for bacteriocins and biosurfactants productions has been described.

## 2. Materials and Methods

### 2.1. Isolation of Lactic Acid Bacteria

Various strains of LAB were isolated from raw milk by enrichment in 100 mL of sterile minimal medium (MM) with 2% paraffin as carbon source. The suspension was incubated at 37°C for 48 h in incubator cum shaker (NSW, India) and it was subcultured on MRS medium [15]. Further, all these isolated cultures were subjected to series of physiological, biochemical, and species-specific standard identification tests [16]. The isolates were stored at −20°C in MRS broth containing 20% (v/v) glycerol stock as master stock until they were further used. The isolate MRTL3 was chosen for further studies.

### 2.2. Biosurfactants and Bacteriocins Extraction

The isolate was grown in MRS broth at 37°C for 72 h. The cell free supernatant (CFS) was adjusted to pH 6.5 with 5 M HCl, heated at 100°C for 3 min, and centrifuged at 10,000 g for 15 min at 10°C for the recovery of bacteriocins. On the other hand, biomass was washed twice with demineralized water, centrifuged (10,000 g, 15 min, 10°C), resuspended in a volume of phosphate buffer saline (PBS; pH 7.0), incubated for 2 h at room temperature, and centrifuged at (10,000 g, 15 min, 10°C) to take the PBS extract free of biomass.

### 2.3. Biosurfactants Production

Biosurfactants produced by the isolate MRTL3 were determined by measuring the surface tension (ST) of the culture supernatant in case of the excreted biosurfactants and of the PBS extracts in the case of the cell-bound biosurfactants. For the recovery of the cell-bound biosurfactants, fermentation medium was centrifuged (10,000 g, 15 min, 10°C) to recover the cells that were washed twice with demineralized water and resuspended in PBS (pH 7.0), incubated for 2 h at room temperature, and centrifuged (10,000 g, 15 min, 10°C). ST of sample was measured by the Ring method [17] using a Tensiometer (Lauda, Germany) equipped with a 1.9 cm De Nouy platinum ring at room temperature. About 10 mL sample was withdrawn every 12 h and surface tension was measured.

### 2.4. Bacteriocins Production

The pH of the samples was adjusted to 6.5 with 5 M NaOH, heated at 100°C for 3 min, and centrifuged (10000 g, for 15 min at 10°C). The presence of bacteriocins in the extracts was qualitatively determined by agar well diffusion assay method against* Staphylococcus aureus* (ATCC 6538P),* S. epidermidis* (ATCC 12228), *Shigella flexneri* (ATCC 9199), *Salmonella typhi* (MTCC 733), *Pseudomonas aeruginosa* (ATCC 15442), *Bacillus cereus* (ATCC 11770), *Listeria monocytogenes *(MTCC 657), and *L. innocua *(ATCC 33090) grown at 37°C in brain heart infusion (BHI; Himedia, India). The antibacterial activity in CFS was determined by well diffusion method [17] with slight modification. CFS of overnight (16–18 h) culture of *L. casei* MRTL3 grown in MRS broth at 37°C was obtained by centrifugation (10,000 g, 15 min, 4°C) and the pH was adjusted to 6.5. To avoid proteolytic degradation of the bacteriocin, CFS was boiled for 3 min. Soft nutrient agar (0.8%, w/v) was allowed to cool down in sterile Petri dish after addition of test organism culture, grown up to the early stationary phase. Wells were made in the lawn of hardened soft agar and aliquots of 50 *μ*L of supernatant were poured in the wells. After 24 h of incubation at the optimal growth temperature of indicator strain, a clear zone of inhibition of at least 2 mm in diameter around cut wells was recorded as positive [18]. Additionally, the antibacterial activity of the biosurfactants produced was also determined by the same procedure.

### 2.5. Colony PCR (16S rRNA Gene Amplification)

Colony PCR of the isolate was performed according to the Sheu et al. [19]. The optimized colony PCR reaction mixture contained 1X PCR amplification buffer (20 mM (NH_4_)_2_SO_4_, 72.5 mM Tris/HCl, 0.1% Tween-20, and pH 9.0), 2.5 mM MgCl_2_, 200 *μ*M each deoxynucleotide triphosphate, 2.5 *μ*M each primer (27f 5′-AGAGTTTGATCMTGGCTCAG-3′ and 1385r 3′-AATTCAAATTTAATTTCTTTCC-5′), and 1.25 U DNA polymerase in 50 *μ*L PCR reaction mixture. Colonies (approximately 1 mm in diameter) were picked up with a sterilized toothpick and directly transferred to the PCR tubes as DNA templates. The thermal cycle programme, run on a thermocycler PCR system (Eppendorf, Germany) consisted of one cycle of 94°C for 10 min, 51°C for 2 min, 72°C for 2 min, and 35 cycles of 94°C for 20 s, 57°C for 45 s (decreased by 1 s per cycle), and 72°C for 1 min, and then incubation at 72°C for 5 min, and a final incubation at 4°C.

### 2.6. Detection of PCR Products

PCR-amplified DNA fragments were observed by Agarose gel electrophoresis in 1.3% Agarose gel in TAE buffer (0.04 M Tris acetate, 0.02 M acetic acid, and 0.001 M EDTA), containing 1 g/mL of SYBR green. Briefly, 10 microlitres of each amplification mixture and the molecular mass marker were subjected to Agarose gel electrophoresis and SYBR green staining. The amplified DNA fragments were visualized by UV illumination.

### 2.7. Sequencing and Analysis of 16S rRNA Gene

The ABI Prism Big Dye Terminator Cycle Sequencing Ready Reaction Kit (Applied Biosystems) was used for the sequencing of the PCR product. A combination of universal primers was chosen to sequence the gene sequence. Samples were run on an ABI PRISM 3730XL DNA Analyzer (Applied Biosystems). Each alignment was checked manually, corrected, and then analyzed using the UPGMA method [19]. Phylogenetic tree was constructed using the MEGA 5 (Molecular Evolutionary Genetics Analysis) software [20, 21].

### 2.8. Product Characterization

#### 2.8.1. Thin Layer Chromatography

Samples were dissolved in ethyl acetate and applied to dried precoated silica TLC plates (Merck, India). Briefly, biosurfactant molecules were prior extracted as follows. Aliquot (4 mL) of CFS was extracted twice with ethyl acetate (Rankem, India) 1 : 1.25 by vigorous vortexing for 2 min. Then upper phase was extracted two times with ethyl acetate and the ethyl acetate was evaporated at room temperature [22, 23]. The extracted product was characterized by using analytical TLC, carried out on Silica gel plates. Briefly, one mL aliquot of each crude biosurfactant sample was concentrated, resuspended in 5 *μ*L of ethyl acetate, and separated on a precoated silica gel plate (Merck, India) using chloroform/methanol/glacial acetic acid (65 : 15 : 2, v/v) as developing solvent system with different post-color-developing reagents. The sugar moieties were stained with anisaldehyde, whereas the fatty acid moieties were stained with ammonium molybdate/cerium sulfate [24, 25].

#### 2.8.2. Purification of Biosurfactant

Crude biosurfactant residue was partially purified in silica gel (60–120 mesh) column eluted with gradient of chloroform and methanol ranging from 20 : 1 to 2 : 1 (v/v). The fractions were pooled after TLC analysis and solvents were evaporated. Once dried, the biosurfactant was stored at 20°C.

#### 2.8.3. Partial Purification of Bacteriocins

CFS from 0.5 L of overnight culture of isolate MRTL3 was prepared as described earlier (Section 2.4). Ammonium sulfate was gently added to the supernatant maintained at 4°C to obtain 60% saturation and the mixture was stirred for 4 h at 4°C. After centrifugation for 1 h at 10,000 g at 4°C, the resulting pellet was resuspended in 30 mL of 25 mM ammonium acetate buffer (pH 6.5) until further use.

#### 2.8.4. Fourier Transform Infrared Spectroscopy (FTIR)

Molecular characterization was performed with a crude biosurfactant sample, which was dialyzed against demineralized water at 4°C in a dialysis membrane (molecular weight cutoff 10,000 kDa, Himedia, India) and then freeze-dried. Characterization was carried out by FTIR analysis of the biosurfactant by scanning it in the range of 4000–400 cm^−1^ at a resolution of 4 cm^−1^ (Model-ABB and MB-3000).

#### 2.8.5. NMR Spectroscopy

The purified biosurfactant was dissolved in deuterated chloroform and 1H analysis was carried out using a Bruker 300 spectrometer. The biosurfactant was dissolved in deuterated chloroform (50 mg mL^−1^) and the spectrum was recorded. 1H chemical shifts were expressed in ppm relative to the solvent shift as chemical standard.

### 2.9. Statistical Analysis

All the values were recorded in triplicates and were subjected to an analysis of standard deviation by using SPSS (v 16) statistical software package.

## 3. Results and Discussion

### 3.1. Screening Assays for Biosurfactant Production

**Figure 1:**
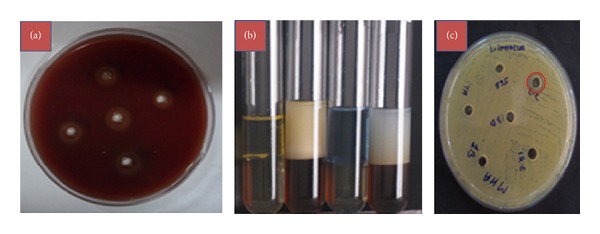
Biosurfactant and bacteriocin production. (a) Hemolysis on blood agar plates. (b) Emulsification of kerosene and diesel oil by culture supernatant. (c) Bacteriocin activity against *Listeria innocua*.

Initially the isolate was screened for its ability to produce biosurfactant and bacteriocin using various qualitative and quantitative methods. As the strain was spot inoculated over the blood agar plate, it resulted in a significant zone of clearance around the colony confirming the production of biosurfactants. In case of drop collapsing test, the flattened drops of CFS over the oil coated surface also indicated the presence of biosurfactant. A clear halo zone (<3 cm^2^) was observed in the oil displacement test and had significant emulsification activity against kerosene oil (Table 1, Figure 1).

### 3.2. Biosurfactant and Bacteriocin Production

In current study, *Lactobacillus casei* MRTL3 was tested for biosurfactant and bacteriocin production using different qualitative and quantitative methods (Figure 1). *Lactobacillus casei* MRTL3 was found positive for cell-bound and excreted biosurfactant and extracellular bacteriocin production. The crude bacteriocin and biosurfactant isolated from strain *Lactobacillus casei* MRTL3 showed significant antimicrobial activity against a broad range of pathogens, including gram positive (*Staphylococcus aureus* ATCC 6538P,* S. epidermidis* ATCC 12228, *Bacillus cereus* ATCC 11770, *Listeria monocytogenes *MTCC 657, and *L. innocua *ATCC 33090) and gram negative bacteria (*Shigella flexneri* ATCC 9199, *Salmonella typhi* MTCC 733, and *Pseudomonas aeruginosa* ATCC 15442). Several biosurfactants and bacteriocins that exhibit antimicrobial activity have been previously described [12], but their simultaneous production has not been reported earlier. Gudina et al. [26] have suggested various approaches for successful recovery of biosurfactant and bacteriocin under different pH conditions using *Lactococcus lactis* and they recovered both the product separately. Antibacterial properties of biosurfactants and bacteriocins isolated from *Lactobacillus casei* MRTL3 against pathogenic bacteria were found similar to those obtained with the crude biosurfactant recovered from *Streptococcus thermophilus* with various concentrations (25 to 100 mg mL^−1^). The surface tension of the production media was reduced to 40.8 mN/m from its initial value of 53 mN/m (Table 2). This decrease of surface tension confirmed the production of biosurfactants by the isolate and its accumulation within the media. Such a reduction in surface tension during the logarithmic and stationary phase has already been reported for various biosurfactant producing microorganisms [27, 28].

Decrease in surface tension of the culture broth was observed for all the strains after 72 h of incubation, although those reductions varied markedly from 9 to 14 mN/m when compared to the surface tension of MRS broth (53.0 mN/m). The ability of a biosurfactant to bring down the surface tension of water from 72.4 to 40.8 mN/m is considered to be a good characteristic of a potent surface active agent. The biosurfactant production was found to be growth associated, as a parallel relationship was observed between biomass production and the decrease in culture broth surface tension. Similar results have been reported by Gudina et al. [26] while using different lactobacilli for the production of biosurfactants under optimal conditions. The lowest value of surface tension was obtained at the end of the shake flask experiment. The strain MRTL3 showed the highest excreted biosurfactant production rate with a reduction in the culture broth surface tension from 53.0 to 40.8 mN/m. The growth and biomass were restricted due to high yield of lactic acid production during the shake flask experiments. The culture supernatant responded positively to oil collapse method and emulsification capacity, and reduction of surface tension was observed.

### 3.3. TLC Analysis

**Figure 2:**
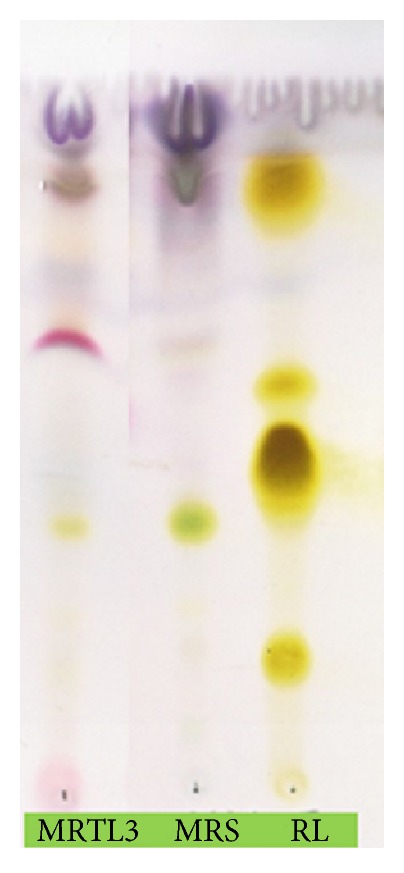
Anisaldehyde staining of BS TLC plate.

Separated compounds were detected on the plate as a single pink spot (Figure 2) and dark blue spots for fatty acids. It confirmed the presence of glycolipid biosurfactant.

### 3.4. Molecular Characterization of the Isolate MRTL3 Using 16S rRNA Sequencing

Amplified DNA fragments were sequenced using Sanger Dideoxy method [29]. Forward and reverse sequence of isolate was joined using DNA Baser v 3.5.3 software and finally was identified as *Lactobacillus casei*. The 16S rRNA gene sequence obtained from the isolate was compared with other bacterial sequences by using NCBI mega BLAST (http://blast.ncbi.nlm.nih.gov/Blast.cgi) for their pairwise identities. Isolate has shown 99% identity with *Lactobacillus casei* ATCC 334 with maximum query coverage of 98%. Further, Consensus sequences were aligned and compared with the available database in NCBI. The 16S rRNA sequence of the isolated bacterium was submitted in the Genbank (NCBI) with an accession number KC568563.

### 3.5. FTIR Spectrometry Analysis

**Figure 3:**
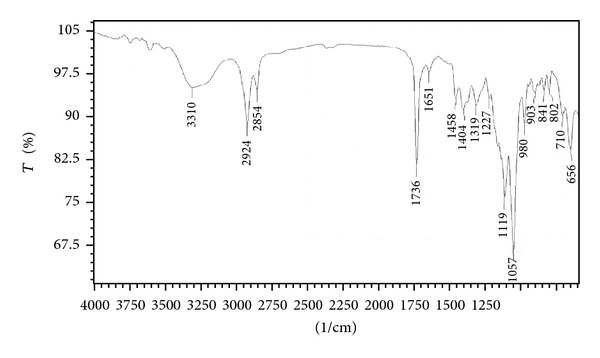
FTIR absorption spectra of partially purified biosurfactant produced by strain MRTL3.

The molecular composition of the partially purified biosurfactant was determined by FTIR spectroscopy (Figure 3) which reveals the presence of carbohydrate and lipid combination. The most significant bands were located 1736 cm^−1^ (for the C=O ester bond) and 1057 cm^−1^ (polysaccharides), 3310 cm^−1^ confirming OH stretching, and 1400–1460 cm^−1^ for C=H stretching which confirmed the presence of glycolipid moieties. The compound showed the C–H stretching vibrations in the transmittance range 2924 cm^−1^ indicating the aliphatic chain.

### 3.6. NMR Analysis

**Figure 4:**
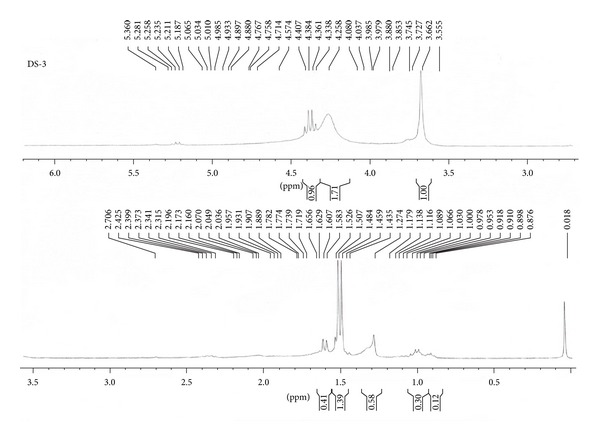
1H NMR spectrum of biosurfactant produced by *Lactobacillus casei* MRTL3.

The data of 1H-NMR spectrum of compound (Table 3; Figure 4) indicate that the compound was a glycolipid. Proton NMR analysis confirmed the presence of lipid and sugar moiety in the biosurfactant. The presence of the methyl esters in the structure of biosurfactant can be related to an increase of its hydrophobicity and, consequently this leads to the increment of their surfactant powers and antifungal and hemolytic activities.

### 3.7. Antibacterial Property of Bacteriocin and Biosurfactant

The antibacterial activity of bacteriocin and biosurfactants produced by the isolate *L. casei* MRTL3 was tested against 8 pathogenic cultures. The results show that the culture exhibits antibacterial activity against these food-borne pathogens (Table 4).

### 3.8. Phylogenetic Relationship

**Figure 5:**
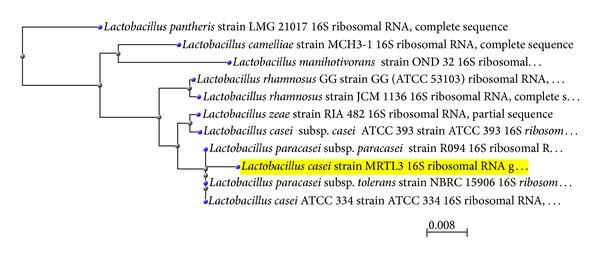
Phylogenetic relatedness of *Lactobacillus casei* MRTL3 using UPGMA tree.

The phylogenetic tree was constructed using MEGA 5.05 software to determine evolutionary relationships of the isolate (Figure 5).

## 4. Conclusion

In the present study, isolate (*Lactobacillus casei *MRTL3) possessing simultaneous bacteriocin and biosurfactant-producing ability was identified and characterized. *Lactobacillus casei* MRTL3 shows remarkable ability to decrease surface tension and also shows inhibitory effect against various food-borne pathogens. Further, the structural characteristics were determined using TLC, FTIR, and 1H NMR spectroscopy which confirmed that the compound is a glycolipid.

Thus, the result obtained suggests that the isolate *L. casei* MRTL3 is a potent lactic acid bacterium as a biosurfactant and bacteriocin producer and possibly can be used as the source of biopreservatives (bacteriocin and/or biosurfactant) in the food processing sector, being a suitable alternative to chemical/conventional preservatives.

## Figures and Tables

**Table 1:** Testing of MRTL3 for biosurfactant production by various methods.

Strain(s)	Surface tension		Emulsification index		Hemolysis
	CFS*	PBS extract**	Kerosene	Diesel	
MRTL3	46.8 ± 0.15	40.8 ± 0.20	58 ± 0.15	57 ± 0.20	++

*Surface tension of MRS is 53.0 mN/m; **surface tension of PBS is 72.4 mN/m.

**Table 2:** Surface tension values (mN/m) of the crude biosurfactant produced by MRTL3.

Strain(s)	Surface tension (mN/m)*						
	0 h	12 h	24 h	36 h	48 h	60 h	72 h
MRTL3	53.1 ± 0.05	40.8 ± 0.15	40.7 ± 0.26	40.9 ± 0.10	41.2 ± 0.10	41.1 ± 0.17	41.4 ± 0.05

*MRS surface tension was 53.0 mN/m.

**Table 3:** Chemical shift assignment of biosurfactant in 1H NMR.

Assignment	Chemical shift (ppm)
CH_3_–(CH_2_)_*n*_–	0.918
–(CH_2_)_*n*_–	1.274
–CH_2_OH	3.662
1-H (sugar)	4.384

**Table 4:** Antibacterial activity shown by bacteriocin and biosurfactant against various test organisms.

Indicator organisms	Origin	Bacteriocin*	Biosurfactant*
*Pseudomonas aeruginosa *	ATCC 15442	+++	+++
*Salmonella typhi *	MTCC 733	+++	+++
*Shigella flexneri *	ATCC 9199	+++	+++
*Staphylococcus aureus *	ATCC 6538P	++++	++++
*Staphylococcus epidermidis *	ATCC 12228	++++	+++
*Listeria monocytogenes *	MTCC 657	++++	++++
*Listeria innocua *	ATCC 33090	++++	++++
*Bacillus cereus *	ATCC 11770	++++	++++

*Excellent >8 mm (++++), good <8mm (+++), and fair <5 mm (++).
